# The Toxic Amblyopias : Their Symptoms, Pathology and Treatment

**Published:** 1896-07

**Authors:** 


THE TOXIC AMBLYOPIAS : THEIR SYMPTOMS, PATHOLOGY AND TREATMENT. By GEO. E. DE SCHWEINITZ, M. D., Clinical Professor of Ophthalmology, Jefferson Medical College, of Philadelphia. Octavo, 240 pages, forty-one engravings and nine full-page colored plates. Limited edition. De luxe binding, $4.00 net. New York and Philadelphia : Lea Brothers & Co., Publishers. 1896.

Within the past few years the different forms of blindness of diverse and unknown nature have gradually decreased in number, until today the amblyopias, especially of toxic origin, are becoming to be well understood and appreciated. Recently three monographs have appeared on the toxic amblyopias, those of Berger, 1892; Knies, 1893, and Casey Wood, 1894, to which is now added the volume before us, known as the Alvarenga prize essay.

De Schweinitz has studied carefully the effect of the different medicinal and toxic agents upon the terminal nerve fibers of the optic nerve, and has produced a most interesting and instructive essay. Of course, the bulk of the work pertains to the amamotic changes following the chronic use of alcohol and tobacco. The real cause of the blindness due to these agents was unknown until Samelsohn's discovery of a retrobulbar neuritis in 1882. Ostensibly the same lesion, interstitial neuritis, is found in other forms of alcoholic neuritis, occurring especially in the legs. Far more often than alcoholic, nicotine produces at times very acute symptoms of amblyopia, and the many instances and references given by the author are of much interest. The Turks and Spaniards, (Island of Cuba,) seem to enjoy a certain immunity from tobacco amblyopia, and withstand the evil influence of excessive inhalation of smoke. In the following chapters are discussed the effects of bisulphide of carbon, iodoform, the coal tar products, arsenic, lead, the anesthetics, caffein, thein, quinine, salicylic acid, the mydriatics and myotics, filix mas, ptomaines, meat-fist and sausage poisoning, serpent virus and the relation of hysteria to certain varieties of toxic amblyopia.

The visual fields accompany the cases reported by the author, while full-plate engravings of the neurotic changes in the optic nerve form an important feature of the work. The bibliography is unusually full and complete—care having been taken to have it verified. On the whole the subject has been masterly treated by the author, and must stand as a valuable addition to this nebulous and long misunderstood subject.

No less attractive is the beautiful manner in which the publishers have embellished the work. W. C. K.

